# The severity assessment of Parkinson's disease based on plasma inflammatory factors and third ventricle width by transcranial sonography

**DOI:** 10.1111/cns.14670

**Published:** 2024-03-08

**Authors:** Yue Lu, Wenwen Dong, Xingya Xue, Jian Sun, Jiuqi Yan, Xiang Wei, Lei Chang, Liang Zhao, Bei Luo, Chang Qiu, Wenbin Zhang

**Affiliations:** ^1^ Department of Functional Neurosurgery The Affiliated Brain Hospital of Nanjing Medical University Nanjing China; ^2^ Department of Neurology Northwest University First Hospital Xi'an China

**Keywords:** cystatin C, LASSO logistic regression, Parkinson's disease, transcranial sonography

## Abstract

**Background:**

Predicting Parkinson's disease (PD) can provide patients with targeted therapies. However, disease severity can be roughly evaluated in clinical practice based on the patient's symptoms and signs.

**Objective:**

The current study attempted to explore the factors linked with PD severity and construct a predictive model.

**Method:**

The PD patients and healthy controls were recruited from our study center while recording their basic demographic information. The serum inflammatory markers levels, such as Cystatin C (Cys C), C‐reactive protein (CRP), RANTES (regulated on activation, normal T cell expressed and secreted), Interleukin‐10 (IL‐10), and Interleukin‐6 (IL‐6) were determined for all the participants. PD patients were categorized into early and mid‐advanced groups based on the Hoehn and Yahr (H‐Y) scale and evaluated using PD‐related scales. LASSO logistic regression analysis (Model C) helped select variables based on clinical scale evaluations, serum inflammatory factor levels, and transcranial sonography measurements. The optimal harmonious model coefficient λ was determined via 10‐fold cross‐validation. Moreover, Model C was compared with multivariate (Model A) and stepwise (Model B) logistic regression. The area under the curve (AUC) of a receiver operator characteristic (ROC), brier score, calibration curve, and decision curve analysis (DCA) helped determine the discrimination and calibration of the predictive model, followed by configuring a forest plot and column chart.

**Results:**

The study included 113 healthy individuals and 102 PD patients, with 26 early and 76 mid‐advanced patients. Univariate analysis of variance screened out statistically significant differences among inflammatory markers Cys C and RANTES. The average Cys C level in the mid‐advanced stage was significantly higher than in the early stage (*p* < 0.001) but not for RANTES (*p* = 0.740). The LASSO logistic regression model (λ.1se = 0.061) associated with UPDRS‐I, UPDRS‐II, UPDRS‐III, HAMA, PDQ‐39, and Cys C as the included independent variables revealed that the Model C discrimination and calibration (AUC = 0.968, Brier = 0.049) were superior to Model A (AUC = 0.926, Brier = 0.079) and Model B (AUC = 0.929, Brier = 0.071) models.

**Conclusion:**

The study results show multiple factors are linked with PD assessment. Moreover, the inflammatory marker Cys C and transcranial sonography measurement could objectively predict PD symptom severity, helping doctors monitor PD evolution in patients while targeting interventions.

## Graphical abstract

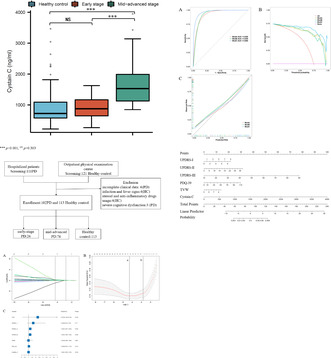

## 1 INTRODUCTION

Parkinson's disease (PD) is a progressive and neurodegenerative disease with motor and nonmotor manifestations, often endangering the elderly.^1^, ^2^ Due to the lack of curative treatments, identifying significant and sensitive biomarkers that could predict PD diagnosis and progression is quintessential.^3^, ^4^ The methods to evaluate the symptoms and signs of PD in clinical practice already exist. However, they are relatively single and limited, and the scale relies heavily on the experiences of neurologists and lacks objective evaluation indicators. Moreover, the positive effect of dopaminergic drugs could affect the PD symptom assessment.^5^

Clinically, the Unified Parkinson's Disease Rating Scale (UPDRS) is primarily used to define PD severity and categorize patients.^6^ Comprising five parts, UPDRS considers motor, nonmotor symptoms and motor complications of PD patients which help determine the stages and clinical severity. The Hoehn and Yahr (H‐Y) stage is a relatively responsive marker of overall PD symptom severity on antiparkinsonian treatment.^7^, ^8^ According to the Hoehn and Yahr scale, PD patients can be categorized into early and mid‐advanced stages.^9^, ^10^ However, clinical scales can roughly assess the clinical manifestations and symptom severity without continuously tracking PD progression. Furthermore, evaluative scales in clinical practice are subjective. To ensure the accuracy and reliability of the assessment results, it must be performed by professional neurologists. Using the scales in PD patients' daily lives is prone to certain limitations.^11^, ^12^

Evidence suggests that neuroinflammation is crucial in PD onset and progression. Thus, high levels of peripheral inflammatory mediators and low levels of anti‐inflammatory cytokines are associated with faster progression of PD.^13^ Due to the significant bidirectional crossover between peripheral and central inflammation, peripheral inflammatory blood markers could be readily available and assess PD severity. Metabolomics of biofluids is promising in identifying biomarkers for early diagnosis and assessing symptom severity in PD patients.^14^ Peripheral venous blood contains various metabolic final products and is an ideal source of rich metabolites serving as biomarkers for the occurrence and development of neurological diseases.^15^

In recent years, several studies have described the involvement of serum Cystatin‐C (Cys C) in degenerative disease progression.^16^ Serum Cys C, a cysteine protease inhibitor C synthesized by nucleated cells, widely exists in all body fluids. However, Cys C is unrelated to gender, diet, or body surface area and is synthesized and released uniformly inside the blood, rendering it an ideal laboratory marker.^17^, ^18^ Cys C is a sensitive laboratory index used to assess renal function and could be linked with neurological diseases. Thus, detecting Cys C levels in specific tissues and body fluids is significant in identifying disease markers and studying disease progression and treatment effects. Some studies have established that Cys C level in the PD cohort was significantly higher than in healthy people, which increased gradually with the disease progression and age of PD patients.^19^

Cognitive impairment is a common non‐motor symptom in PD, affecting nearly 42% of patients during initial diagnosis. Almost 80% of PD patients with a disease duration greater than 20 years will develop dementia.^20^ PD combined with cognitive impairment significantly affects individual prognosis, severely declining the quality of life of patients and doubling mortality. Therefore, cognitive impairment is one of the indicators to evaluate PD. Transcranial sonography (TCS)‐assessed third ventricle width (TVW) helps determine global and focal brain atrophy with an accuracy comparable to magnetic resonance imaging.^21^

Our study assessed the motor and neuropsychological testing results, serum inflammatory levels, and transcranial sonography examination across various PD stages. The study's objective helped evaluate the role of serum inflammatory factors and transcranial sonography in differentiating the clinical stages and assessing symptom severity in PD patients. The study provides insights into PD‐related pathogenesis, disease severity prediction, and disease‐targeted interventions.

## 2 MATERIALS AND METHODS

### 2.1 Patient recruitment and clinical assessment

**FIGURE 1:**
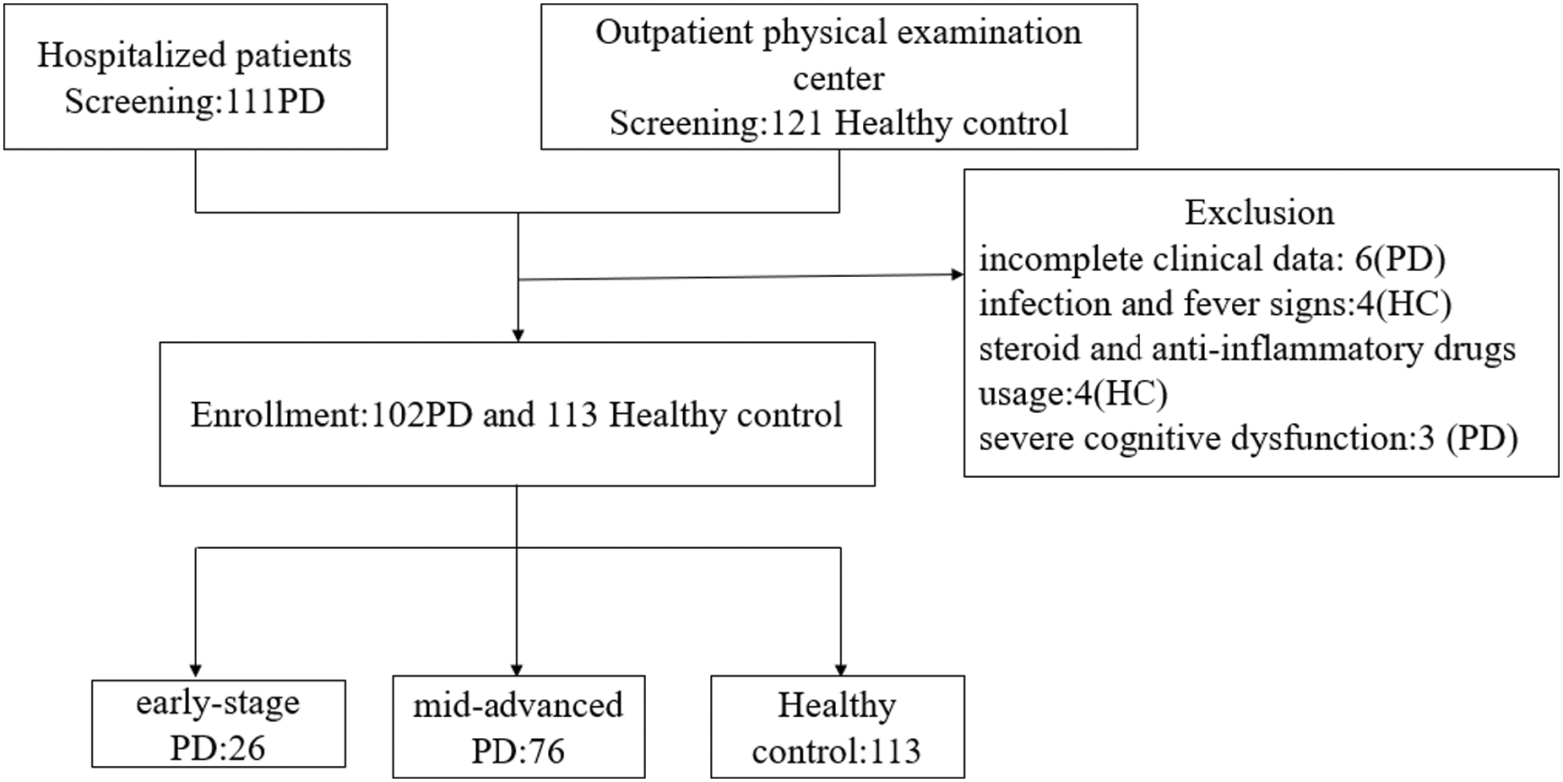
Flow diagram of screening PD patients and healthy controls.

This retrospective study recruited 111 PD patients from The Affiliated Brain Hospital of Nanjing Medical University between October 2017 and October 2019 after obtaining ethical approval from the institutional committee. However, nine were excluded due to incomplete clinical evaluation or severe cognitive dysfunction. The healthy control group (*n* = 113) without neurodegenerative disease symptoms was recruited from the Physical Examination Center of The Affiliated Brain Hospital of Nanjing Medical University (Figure 1). The written information was provided, and informed consent was obtained from the subjects. All the PD patients fulfilled the Movement Disorder Society (MDS) diagnosis criteria and were examined using the Hoehn and Yahr (H‐Y) scale. Based on the H‐Y scale, the PD patients were subdivided into early and mid‐advanced PD. The early‐stage PD group involved 26 patients with the H‐Y stage between 1 and 2. In contrast, the mid‐advanced stage PD group possessed 76 patients with the H‐Y stage between 3 and 5.

The inclusion criteria showed (i) no infection and fever signs and (ii) no use of steroid and anti‐inflammatory drugs 3 months before study recruitment. Exclusion criteria included (i) severe cognitive dysfunction, including dementia, anxiety, and depression; (ii) previous surgical treatment of deep brain stimulation (DBS), thalamotomy, and stem cell therapy; (iii) inability to cooperate with clinical assessment.

Clinical history and scale examinations such as gender, age, disease duration, UPDRS part I, part II, and part III, Montreal Cognitive Assessment (MoCA), Mini‐Mental State Examination (MMSE), Hamilton Anxiety Scale (HAMA), Hamilton Depression Scale (HAMD), and the 39‐item Parkinson's Disease Questionnaire (PDQ‐39) scores were recorded for the PD subjects. Before the scale assessment day, all the patients were instructed to stop taking anti‐parkinsonism medication for at least 12 h. Moreover, dopamine was stopped for 12–24 h, and dopamine agonists were over 3 days.

### 2.2 Blood sample collection and examination

Peripheral venous blood samples were obtained from all the PD patients and healthy controls while resting in the morning. One blood sample tube was sent to the clinical laboratory center for immediate analysis and routine biochemical parameter measurement. This included neutrophil counts, lymphocyte counts, lymphocyte/neutrophil ratios, lactate dehydrogenase, lipoproteins, and blood glucose levels. The other tube of blood samples was kept inside heparin tubes and centrifuged for 15 min at 3000 rpm to obtain the plasma samples. Then, the samples were stored at −80°C until further analysis.

Cys C, CRP, RANTES, IL‐10, and IL‐6 plasma levels were measured using Luminex kits from Millipore (Billerica, USA) following the manufacturer's instructions. The procedure involved diluted plasma samples incubated using antibody‐coupled microspheres. This was followed by incubation with biotin‐detecting antibodies and by adding streptavidin‐alginin. The instrument parameters of the FLEXMAP 3D system (Luminex Corporation, Austin, USA) were set to events/bead, 50; sample size, 50 μL; discriminator gate, 8000–15,000. Then, the raw data were measured and obtained using xPONENT 5.1 software. Later, the raw data were imported within the Milliplex Analyst 5.1 software (VigeneTech, USA). This was followed by measuring the plasma protein concentration levels with a 3‐parameter logistic fit.

### 2.3 Third ventricle width measurement

The TVW was measured by TCS in all PD patients. Two experienced sonographers unaware of the clinical stages of PD patients performed TCS to ensure the accuracy and reliability of the measurement. The transcranial sonography images were stored and uploaded to the cloud and deciphered by two other sonographers, focusing on the structure of the brain nucleus. They determined whether the blood flow of the cerebral arteries was abnormal while measuring the TVW. An ultrasound device (PHILIPS EPIQ 7) equipped with a 2.5 MHz phased array transducer was utilized for every study subject. An ultrasound brain image was conducted via the preauricular transtemporal bone window, which had a penetration depth of 14–16 cm and a dynamic range of 45–55 dB. The butterfly shaped mesencephalon, starting from the brainstem plane, was demonstrated. Then, the ventricular plane was visualized by tilting the probe upwards about 10°–15°. At this plane, the third ventricle could be identified as two parallel hyperechogenic lines within the image center where TCS measurements were performed from the ipsilateral to the contralateral inner layer of the hyperechogenic ependyma.

### 2.4 Statistical analysis

All the data were statistically analyzed using SPSS 25 (IBM) and R software (version 4.3.1). First, continuous data were tested with the Shapiro–Wilk test for normality. Quantitative data following normal distribution were characterized using mean ± standard deviation (SD), and between‐group comparisons were performed using the Student's *t*‐test. Quantitative data not following normal distribution were indicated by the median (interquartile range, IQR), and the Mann–Whitney *U* test helped compare between groups. Then percentages helped determine the categorical data, and Pearson's chi‐squared or Fisher's exact tests helped undergo between‐group comparisons. One‐way analysis of variance (ANOVA) was used when considering healthy controls for comparative analysis. After univariate analysis, the relevant factors were used as independent variables with *p* < 0.05 and the H‐Y stage as dependent variables. Then, multivariate and stepwise logistic regression analyses helped screen meaningful variables and establish a PD severity assessment model. A LASSO logistic regression model utilized the “glmnet” software package to determine the factors linked with PD symptom severity. The cross‐validation method helped choose the reconciliation parameter λ. Then, the variables were subjected to LASSO logistic regression analyses to adjust for the confounding factors. Depending on clinical scales, serum inflammatory indicators, and transcranial sonography examination, the prediction model was established and was compared with multivariate and stepwise logistic regression models. The area under the curve (AUC), Brier score, and calibration curve helped evaluate the discrimination and accuracy of the prediction model. The “forestplot” and “rms” software packages helped construct forest plots, nomogram plots, and calibration curves of LASSO logistic regression models. All the statistical tests were two‐sided, with *p* < 0.05 considered statistically significant.

## 3 RESULTS

### 3.1 Demographics and clinical data

A total of 102 patients with PD and 113 healthy controls were included in the study. The demographic and clinical data of the participants are summarized in Table 1. The mean age of all the PD patients was 63.0 (58.0, 68.0) years, and the mean disease duration was 8.0 (5.0–12.0) years, of which 57.8% were male. Based on the H‐Y stage classification standard, 26 (25.5%) patients were classified as early PD (grade 1–2) and 76 (74.5%) mid‐advanced PD (grade 3–5) patients. Based on the demographic data, no significant differences could be observed in gender and age among different groups. However, a substantial difference was observed in TVW and duration. The median disease duration was more significant in the mid‐advanced stage (8.0 years (6.0–13.0)) than in the early stage (4.0 years (3.0–8.0)) (*p* < 0.001). Significant differences were observed between the early and mid‐advanced stage groups in UPDRS‐I, UPDRS‐II, UPDRS‐III, MMSE, HAMA, HAMD, and PDQ‐39 scores. Thus, all the above indexes were statistically associated with PD symptom severity (*p* < 0.001). Cys C and RANTES were statistically different among the measured biochemical and inflammatory marker levels between the three groups. The mean levels were significantly higher in the mid‐advanced stage group than the healthy controls. However, no significant difference was observed between the early‐stage and healthy control groups. The average level of Cys C is significantly higher (*p* < 0.001) in the mid‐advanced stage than in the early stage but not for RANTES (*p* = 0.740). Therefore, increasing Cys C levels could be associated with PD symptom severity and clinical staging.

**TABLE 1:** Comparison of clinical and inflammatory markers between healthy controls and PD patients.

	Controls (*n* = 113)	Early stage (*n* = 26)	Mid‐advanced stage (*n* = 76)	*p*
Gender				
Female	45 (39.8%)	8 (30.8%)	35 (46.1%)	0.258
Male	68 (60.2%)	18 (69.2%)	41 (53.9%)	
Age (years)	63.0 (56.0–68.5)	65.0 (57.5–67.8)	63.0 (58.0–69.2)	0.857
TVW		6.00 (0.45)	6.75 (0.57)	<0.001***
Duration (years)	–	4.0 (3.0–8.0)	8.0 (6.0–13.0)	<0.001***
UPDRS‐I	–	2.0 (1.0–3.0)	5.0 (3.0–7.0)	<0.001***
UPDRS‐II	–	10.0 ± 5.28	16.2 ± 5.42	<0.001***
UPDRS‐III	–	29.8 ± 8.33	50.9 ± 12.6	<0.001***
MoCA	–	25.0 (23.2–27.0)	23.5 (20.0–26.0)	0.105
MMSE	–	28.0 (25.2–29.0)	25.0 (21.8–27.0)	0.012*
HAMA	–	4.00 (2.25–5.75)	10.0 (6.00–12.0)	<0.001***
HAMD	–	4.50 (4.00–8.00)	11.0 (8.00–13.0)	<0.001***
PDQ‐39	–	36.7 ± 16.5	64.6 ± 18.5	<0.001***
Neutrophils (G/L)	–	3.14 (2.77–3.50)	3.47 (2.87–4.03)	0.192
Lymphocyte (G/L)	–	1.75 ± 0.46	1.64 ± 0.61	0.338
L/N	–	0.54 (0.34–0.70)	0.46 (0.30–0.63)	0.200
LDH (U/L)	–	171 (152–195)	166 (149–186)	0.375
HDL‐C (mmol/L)	–	1.25 ± 0.24	1.29 ± 0.27	0.569
CHOL (mmol/L)	–	4.06 (3.65–4.46)	4.36 (3.92–4.86)	0.106
Glu (mmol/L)	–	5.04 (4.64–5.44)	5.01 (4.63–5.88)	0.730
Cys C (ng/mL)	720 (570–1097)^a^ ^,^ ^b^	886 (644–1160)^c^	1533 (1128–2023)	<0.001***
CRP (ng/mL)	11,703 (5600–38,900)	12,634 (4854–40,145)	13,476 (6381–27,638)	0.881
RANTES (ng/mL)	23.14 (13.82–43.74)^d^ ^,^ ^e^	28.72 (22.28–61.27)^f^	36.18 (23.52–57.63)	0.049*
IL‐10 (pg/mL)	5.21 (0.90–15.7)	6.66 (1.70–27.4)	4.88 (0.66–15.7)	0.367
IL‐6 (pg/mL)	0.00 (0.00–0.07)	0.00 (0.00–0.02)	0.01 (0.00–0.24)	0.229

Abbreviations: CHOL, cholesterol; CRP, C‐reactive protein; Cys C, cystain C; Glu, glucose; HAMA, Hamilton Anxiety Scale; HAMD, Hamilton Depression Scale; HDL‐C, high‐density lipoprotein‐cholesterol; IL, interleukin; L/N, lymphocyte/neutrophil ratios; LDH, lactate dehydrogenase; MMSE, Mini‐Mental State Examination; MoCA, Montreal Cognitive Assessment; PDQ‐39, The Parkinson's Disease Questionniare‐39; RANTES, regulated on activation, normal T cell expressed and secreted; UPDRS, Unified Parkinson's Disease Rating Scale.

^a^ *p* = 0.303 versus early stage.

^b^ *p* <0.001 versus mid‐advanced stage.

^c^ *p* <0.001 versus mid‐advanced stage.

^d^ *p* = 0.073 versus early stage.

^e^ *p* < 0.05 versus mid‐advanced stage.

^f^ *p* = 0.740 versus mid‐advanced stage (Mann–Whitney test).

*p < 0.05, ***p < 0.001.

### 3.2 Multivariate analysis

As shown in Table 2, 10 variables (*p* < 0.05) were included in multivariate logistic regression analysis. The results indicated that the UPDRS‐III score was an independent risk factor for PD symptom severity (*p* < 0.05). Furthermore, stepwise logistic regression analysis revealed that UPDRS‐III and HAMD were significantly linked with PD severity. Two clinical PD process prediction models were constructed, such as the UPDRS‐III (Model A) and UPDRS‐III and HAMD scores (Model B).

**TABLE 2:** Screening the variables related to the severity of PD symptom.

	Multivariate logistic regression OR (95% CI)	Stepwise logistic regression OR (95% CI)	LASSO logistic regression OR (95% CI)
TVW	16.55 (0.116–32.533)		2.275 (0.146–32.45)
Duration (years)	0.828 (0.228–3.011)	–	–
UPDRS‐I	6.918 (1.379–18.8)	8.167 (1.527–9.318)	1.645 (0.84–3.719)
UPDRS‐II	2.482 (1.321–18.19)	2.627 (1.383–14.980)	1.244 (1.027–1.604)*
UPDRS‐III	1.992 (1.276‐8.27)*	1.976 (1.294–6.985)*	1.223 (1.048–1.534)**
MMSE	1.377 (0.5–10.88)	–	–
HAMA	2.256 (0.745–25.89)	2.3 (0.915–13.560)	1.003 (0.598–1.595)
HAMD	0.318 (0.03–0.719)	0.321 (0.047–0.696)*	–
PDQ‐39	1.184 (0.993–1.945)	1.127 (0.999–1.565)	1.058 (0.975–1.174)
Cys C (ng/mL)	1.016 (1.005–1.045)	1.018 (1.006–1.051)	1.005 (1.002–1.012)*

Abbreviations: AIC, Akaike information criterion; BIC, Bayesian information criterion.; Cys C, cystain C; HAMA, Hamilton Anxiety Scale; HAMD, Hamilton Depression Scale; MMSE, Mini‐Mental State Examination; PDQ‐39, The Parkinson's Disease Questionniare‐39; UPDRS, Unified Parkinson's Disease Rating Scale.

*p < 0.05, **p < 0.01.

**FIGURE 2:**
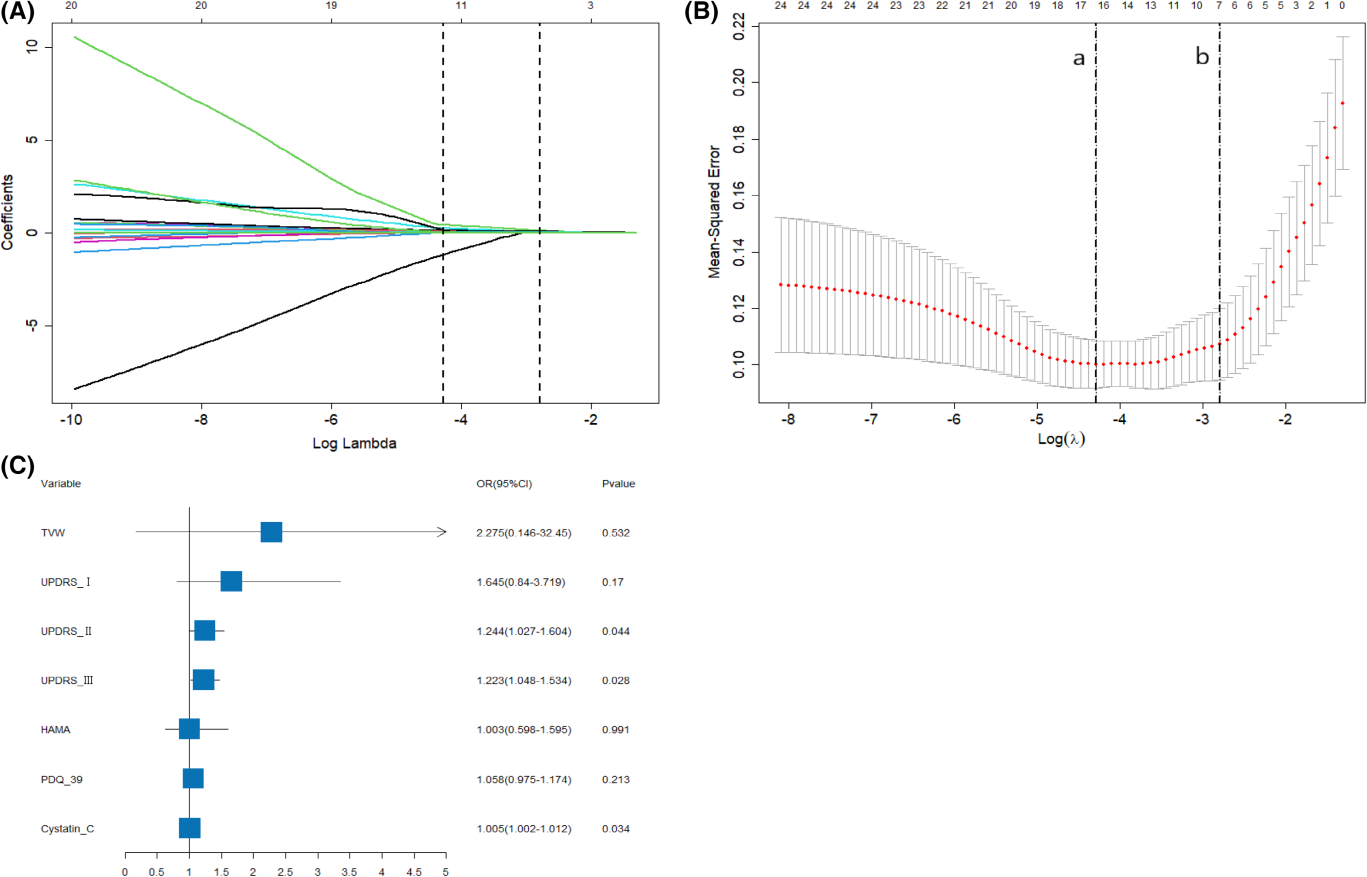
Characteristic variable selection based on LASSO logistic regression. (A) The relationship between log(λ) and the LASSO regression coefficients. (B) The curve of log(λ) versus the number of independent variables. When the optimal λ.1se = 0.061, seven independent variables were filtered out. (C) LASSO logistic regression forest plot.

In addition, LASSO logistic regression analysis helped analyze clinical indicators associated with the PD process, and the screening of the variables was shown as a λ function in Figure 2. Figure 2A represents the relationship between log(λ) and LASSO regression coefficients. As λ increased, the estimated independent variable coefficients were significantly compressed in the model. The coefficients of the independent variables were compressed to 0 with a negligible effect on the dependent variable and reduced the number of independent variables.

Figure 2B depicts the curve of log(λ) versus the number of independent variables, with the model mean‐square error (MSE) in the vertical coordinate and log(λ) in the lower horizontal coordinate. The number of non‐zero coefficient independent variables in the model corresponded to different log(λ) within the upper horizontal coordinate. The dashed line “a” in Figure 2B characterizes the optimal reconciliation coefficient λ.min = 0.014 at the minimum MSE. The dashed line “b” depicts the optimal λ.1se = 0.061 in one standard MSE error. In this study, the optimal model (Model C) was selected at λ.1se =0.061, since the independent variables associated with LASSO logistic regression model were TVW, UPDRS‐I, UPDRS‐II, UPDRS‐III, HAMA, PDQ‐39, and Cys C. The parameter estimates of the LASSO logistic regression are represented in Table 2. The results showed that UPDRS‐II (OR = 1.244, 95% CI: 1.027–1.604), UPDRS‐III (OR = 1.223, 95% CI: 1.048–1.534), and Cys C (OR = 1.005, 95% CI: 1.002–1.012) significantly correlated with PD symptom severity. The LASSO logistic regression forest plot (Figure 2C) helped visualize the factor effect values (OR and 95% CI) and *p*‐values associated with PD symptom severity.

### 3.3 Comparison of models

**FIGURE 3:**
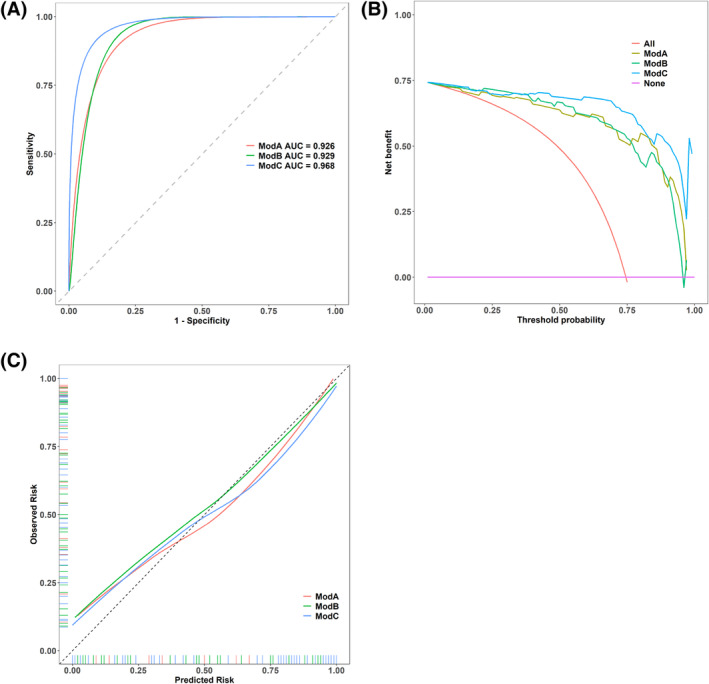
Multiple model comparison. (A) To predict PD progression, the AUCs of the models A, B, and C were 0.926, 0.929, and 0.968. (B) The decision curve analysis of three models was demonstrated. (C) Calibration curves were displayed for three models.

The three models were compared after the discrimination, calibration, and decision curves in Figure 3. For discrimination, the areas under the curve (AUCs) of Model A, Model B, and Model C were 0.926, 0.929, and 0.968, respectively. Thus, the LASSO logistic regression model discriminates better than multivariate and stepwise logistic regression models. The Brier scores helped assess the overall model performance in Model A, Model B, and Model C were 0.079, 0.071, and 0.049, respectively. Thus, based on decision and calibration curves, Model C performed better than Model A and Model B.

**FIGURE 4:**
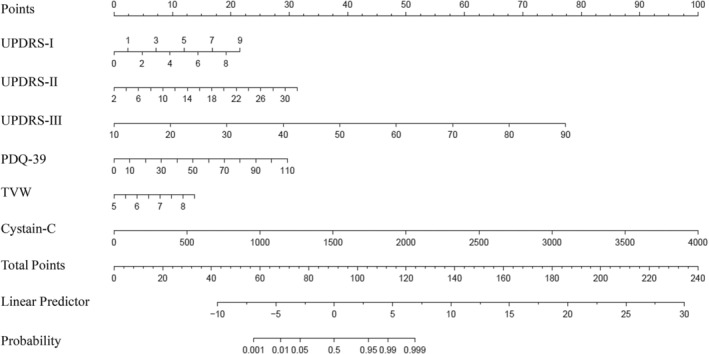
The LASSO logistic regression prediction nomogram.

After adjusting the confounding factors, TVW, UPDRS‐I, UPDRS‐II, UPDRS‐III, PDQ‐39, and Cys C were linked with PD process development. Depending on their influencing roles in predicting the PD process, a LASSO logistic regression prediction nomogram was constructed after the clinical and inflammatory metabolic indexes to predict PD symptom severity (Figure 4).

## 4 DISCUSSION

PD patients inevitably experience disease progression post‐diagnosis. During the late disease stages, patients often suffer from severe complications such as recurrent falls, wheelchair dependence, and severe dementia.^22^ The H‐Y scale is an effective indicator in clinical practice to assess PD symptom severity. Therefore, based on this measure, patients are categorized into early and mid‐advanced stages (H‐Y 1–2 and H‐Y 3–5). However, this assessment requires the expertise of a neurologist providing a rough staging for PD patients. Moreover, the evaluation is limited by its practical application in daily life. Currently, continuous tracking of the disease severity needs to be improved. Researchers such as Goetz and his colleagues infer the presence of significant differences in disease progression rates among PD patients across different H‐Y stages.^10^, ^23^ PD progresses relatively slowly within stages 1 and 2 and can gradually stabilize using oral anti‐Parkinson's medication. However, the disease accelerates once it progresses to stage 3, irrespective of the treatment approach, accompanied by different complications. Therefore, timely identification of the early to mid‐advanced‐stage transition in PD patients provides clinical significance. This can assist neurologists in adopting appropriate measures to delay the process of PD.

Previous research^24^, ^25^ has demonstrated that the occurrence and development of PD are closely associated with multiple mechanisms, such as inflammation, oxidative stress, mitochondrial dysfunction, abnormal protein aggregation, and excessive N‐methyl‐D‐aspartate (NMDA) receptor activation. Homocysteine, uric acid, Cys C, and CRP are implicated in the pathogenesis and progression of degenerative diseases.^26^, ^27^, ^28^ The elevated homocysteine and Cys C levels and lower low‐density lipoprotein cholesterol levels may enhance PD susceptibility.^29^, ^30^

Initially, this study performed univariate analysis to screen for factors linked with PD symptom severity. Subsequently, multivariate and lasso logistic regression analyses were conducted to identify inflammatory markers significantly correlated with the PD severity. Finally, a comprehensive model was constructed depending on clinical scales and biochemical inflammation markers to predict PD symptom severity. The model developed through lasso logistic regression depicted excellent discrimination and calibration performance. The model identified Cys C as an independent risk factor to predict PD symptom severity. Cys C levels gradually increase as the disease severity advances in PD patients. Thus, Cys C is an essential indicator to evaluate PD symptom severity.^13^

Cys C was discovered in human cerebrospinal fluid. However, Cys C was widely present in bodily fluids and tissues, involving various human physiological processes.^31^ Cys C is associated with normal tissue cell proliferation and growth, inflammatory responses, tumor metastasis, and neurodegenerative diseases. Therefore, determining Cys C levels in specific tissues and bodily fluids is essential to identifying disease markers, understanding disease progression, and assessing treatment efficacy.^32^ Nagai et al.^33^ reported that directly injecting Cys C inside the hippocampal neurons of rats could induce death. In contrast, Cys C released by dopaminergic neurons in rats under toxin exposure could activate microglial cells, exacerbating neural damage.^34^ However, some studies demonstrate that Cys C has a neuroprotective role in human neurodegenerative diseases.^17^, ^35^ The potential of Cys C in neuroprotective mechanisms could stimulate the proliferation and mitotic activity of dopamine cells. For instance, Xu et al.^36^ reported that dopaminergic neuronal loss in the midbrain cell cultures of rat fetuses could partially reverse through human Cys C treatment after exposure to 6‐hydroxydopamine. This indicates that the neuroprotective effect of Cys C in PD could be associated with the regeneration of dopaminergic neurons. In PD patients, elevated Cys C levels may inhibit α‐synuclein formation. Still, it could be insufficient to clear abnormal protein aggregates or inhibit Lewy body formation.^37^, ^38^

Oxidative stress is another mechanism via which Cys C exerts a protective effect in PD patients.^39^ During oxidative stress, the body can cause continuous protease expression in intracranial tissues, causing the degeneration and death of dopaminergic neurons while promoting PD development. However, Cys C can act as a protease inhibitor during this period, inhibiting the damage to dopaminergic neurons induced by intracranial tissue proteases. Cys C induces autophagy (a major degradation pathway for misfolded or unfolded proteins and the ubiquitin‐proteasome pathway), degrades alpha‐synuclein, and inhibits aggregation. Moreover, Cys C also regulates vascular endothelial growth factors to enhance vascular formation and dopaminergic neuronal survival.^40^, ^41^ Therefore, increasing Cys C levels or decreasing damage to dopaminergic neurons may significantly improve PD prognosis. The current study observed that Cys C levels were significantly enhanced in mid‐advanced stage PD patients than in the early stages. Therefore, central inflammatory reactions are exacerbated after PD progresses to the late stage. Moreover, the body compensates by elevating Cystatin‐C synthesis to protect dopaminergic neurons from inflammatory damage. However, these findings require further validation.

Non‐motor symptoms are also essential in the comprehensive PD assessment. Emotional disorders usually do not receive enough attention, while PD patients are connected with anxiety and depression comorbidities. Their clinical manifestations overlap with the cognitive impairment and motor features of PD while adversely affecting motor and social performance, decreasing the quality of life. Studies have indicated that anxiety and depression coexist in most PD patients.^20^ Depression was strongly linked with axial motor symptoms, whereas anxiety was more common among younger PD patients and those with motor fluctuations.^42^ Thus, anxiety and depression can indicate the disease severity to a certain extent.

Transcranial ultrasound measurement of the width of the third ventricle became an indicator of cognitive dysfunction among PD patients.^43^ Moreover, the TCS became an assessment tool for cognitive function and disease severity.^44^ Our findings indicate that TCS‐assessed TVW screened by lasso regression was associated with disease severity. Prediction accuracy appears insufficient as a biomarker, and influencing cofactors limit the clinical practice value when used alone. However, it should be considered a screening and evaluation tool while increasing the prediction power associated with other markers.

Our study has certain limitations. First, this is a retrospective study with a small sample recruited from a single center. Second, since this is a cross‐sectional study, more longitudinal studies are required to identify comprehensive and objective indicators to predict PD progression. Lastly, the serum inflammatory marker levels were measured only once among all the participants. Therefore, further studies with larger sample sizes can help decipher how serum inflammatory factors improve PD occurrence and development at cellular and molecular levels.

## 5 CONCLUSIONS

This study observed that the serum Cys C levels of middle‐ and late‐stage PD patients were significantly more elevated than those of early‐stage PD patients and healthy controls. However, no significant difference between early‐stage PD patients and healthy controls was observed in serum Cys C levels. A comprehensive model was constructed to predict PD severity by including serum Cys C, TCS‐assessed TVW, and UPDRS scores in clinical evaluations. The model showed good discrimination and calibration and could help neurologists choose appropriate interventions with high clinical value. Therefore, the inflammatory biomarker Cys C combined with clinical measurements could effectively predict the PD severity.

## AUTHOR CONTRIBUTIONS

YL, WD, XX, and WZ conceptualized the study design. BL, CQ, and WD collected clinical data and analyzed plasma samples. LC, JS, JY, and XW undertook the data analysis. YL, LZ, XX, and WD interpreted the results. YL wrote the manuscript. All authors provided feedback for revision and read and approved the final report.

## CONFLICT OF INTEREST STATEMENT

The authors have no conflicts of interest to declare.

## Data Availability

The data that support the findings of this study are available from the corresponding author upon reasonable request.
